# Comparison of Three Different Types of Two-Implant-Supported Magnetic Attachments on the Stress Distribution in Edentulous Mandible

**DOI:** 10.1155/2019/6839517

**Published:** 2019-04-08

**Authors:** Fengling Hu, Yiming Gong, Zhen Bian, Xiaoying Zhang, Bin Xu, Jianguo Zhang, Xiaojun Shi, Youcheng Yu, Liang Song

**Affiliations:** ^1^Department of Stomatology, The Fifth People's Hospital of Shanghai, Fudan University, 801 Heqing Road, Shanghai 200240, China; ^2^Department of Stomatology, Zhongshan Hospital of Fudan University, 180 Fenglin Road, Shanghai 200032, China; ^3^School of Mechanical Engineering, Shanghai Institute of Technology, Haiquan Road 100, Shanghai 201418, China

## Abstract

Two-implant-retained mandibular overdentures with magnetic attachments can provide an effective treatment modality for edentulous patients. In this study, a three-dimensional finite element analysis was used to compare the biomechanical characteristics of three different types of magnetic attachments in two-implant-retained mandibular overdentures. Flat-type, dome-type, and cushion-type of the magnetic attachments were designed to retain the overdenture. Four types of load were applied to the overdenture in each model: 100 N vertical and oblique loads on the right first molar and a 100 N vertical load on the right canine and the lower incisors. The biomechanical behaviors of peri-implant bone, abutment, and mucosa were recorded. In vertical incisors, vertical right canine, and oblique molar loading condition, the flat-type group exhibited the highest levels of maximum equivalent strain/stress in the peri-implant bone. The total deformation of mucosa and the maximum equivalent strain/stress in the oblique molar loading condition are about two times as the vertical molar loading condition. These results suggested that both cushion-type and dome-type of the magnetic attachments are better choices in two-implant-retained mandibular overdentures, and oblique loading is more harmful than vertical loading.

## 1. Introduction

The mandibular bone resorption is significantly greater than the maxilla. Complete mandibular dentures always have the poor retention problem. Overdenture is a good choice for the mandibular edentulous patients. Dental implants with removable prosthesis are helpful way for rehabilitation of edentulous patients [1]. Magnetic attachments, which have been used to maintain the stability of denture since the 1950s, presented several advantages including long-lasting constant retentive force, reduced lateral forces, and simplicity in installation for patients with dexterity problems [2–4]. They were widely applied in both dental prostheses and implants [5, 6] and have shown high levels of clinical success [7]. Two-implant-retained mandibular magnetic overdentures are stable, cost-effective, and less invasion and have achieved good patient satisfaction [8, 9].

Three types of the magnetic attachments, such as flat-type, dome-type, and cushion-type, are commercially available for implant-retained overdenture. The flat-type is a conventional magnetic attachment that has larger retentive force and greater stress. As new generation of magnetic attachments, the dome-type and cushion-type attachments have the function to allow displacement or rotation of the overdenture during function [10].

Bioengineering tools have been shown to be useful to evaluate the performance of implants and the dentures. Previous studies using strain gauge analyses displayed lower lateral stress distribution for overdentures retained by cushion-type magnetic attachment than did the flat-type [11], while others showed similar effect on the denture movement and lateral stresses between the three types of magnetic attachments [3]. Based on these inconsistent results, it may be better to use other engineering tools to evaluate the biomechanical behaviors of the three magnetic attachment systems.

Three-dimensional finite element method is considered as a precise and effective research approach for investigating stress/strain distribution in the study of prosthodontics [12, 13], which provide precious representation of complex geometries, and the model modification is convenient. When loaded appropriately, the three-dimensional finite element method can reveal the stresses/strains distribution throughout the whole structure [14].

The aim of this study was to evaluate the biomechanical behaviors of three different types of magnetic attachments in two-implant-supported overdentures by three-dimensional finite element analysis methods.

## 2. Materials and Methods

### 2.1. Model Design

**Figure 1:**
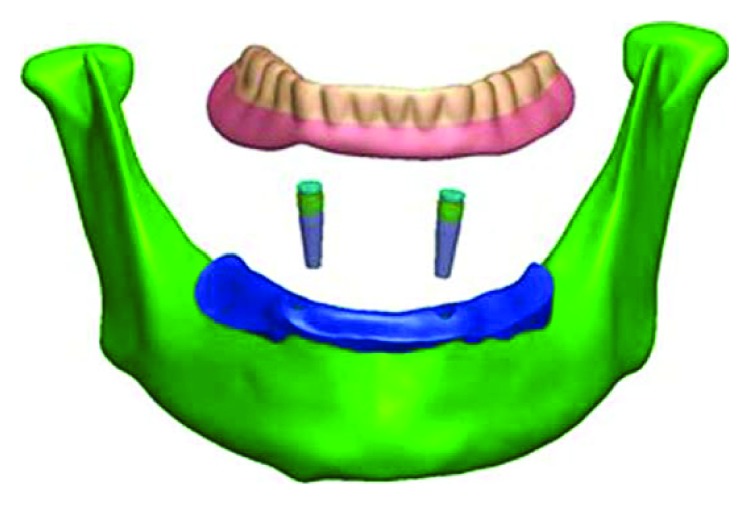
Three-dimensional solid geometric models of mandible, mucosa, overdenture, implants, and magnetic.

**Figure 2:**
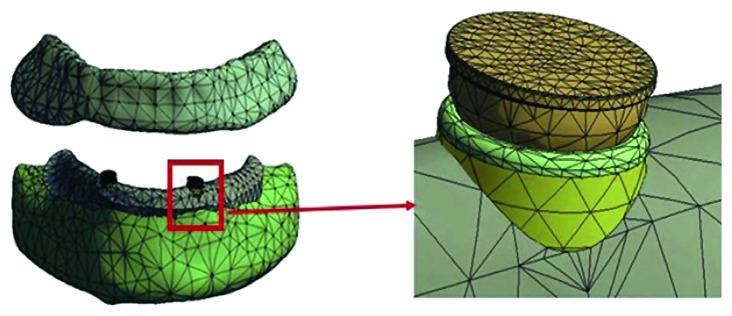
Meshed modeling of jaw, mucosa, and implant magnetic overdenture (flat-type).

The three-dimensional geometry was obtained and acquired through the edentulous mandible, the overdenture, the implant, and the magnetic attachment. Mandibular bone and overdenture computerized tomography (CT) data were obtained from a 63-year-old female volunteer with a complete edentulous mandible covered by a resin complete denture which can provide the precise relationship between the denture and mandible. The CT was done through the KaVo 3D exam (KaVo Dental GmbH, Bismabring, Germany) CBCT scanner, to make the preoperative examination after obtaining the agreement of patient through signing a consent form according to our local Human Research Ethics Committee (#2015EC099) before the surgery. The Digital Imaging and Communications in Medicine (DICOM) data obtained from CT were processed using three-dimensional image processing and editing software (Mimics 10.01, Materialise, Leuven, Belgium). The point cloud data of overdenture, cortical, and cancellous bones were extracted and performed from CT Hounsfield value, using the threshold value and region growth function. Then, modeling software (SolidWorks release 2010, SolidWorks Corporation) was used to transform the reference model subject data into the FEM solid model of the mandibular and the overdenture. The 3D geometry (Figure 1) was exported to FE preprocessing software ANSYS14.1 (ANSYS Inc., Canonsburg, PA, USA) and discretized in linear tetrahedral elements (Figure 2). The mandible surface is assumed a 2 mm constant cortical bone layer wrapped around the cancellous bone surface [15]. Based on the precise location between the mandibular and the overdenture on the CT, the precise geometry of mucosa closely contacted with the inner surface of the denture was obtained [16]. The average thickness of the mucosa covered on the edentulous mandibular was about 2 mm.

**Figure 3:**
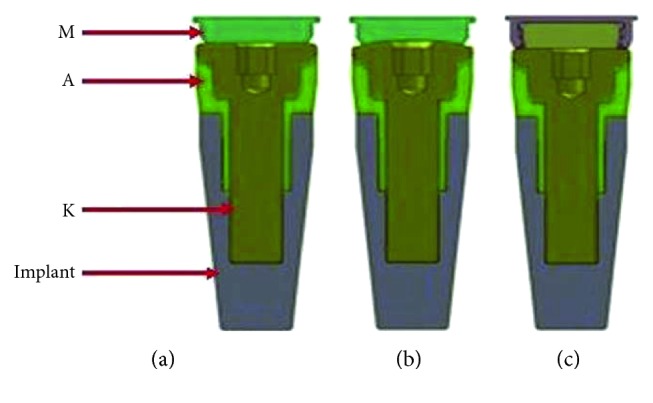
Combination models of the implants and magnetic attachments. The keeper (K) is screwed onto the abutment cylinder (A) and inserted into the implant, and the magnet (M) is assembled in the denture. (a) Flat-type. (b) Dome-type. (c) Cushion-type.

The models of two implants (4.3 mm in diameter, 10.0 mm in length; Nobel Replace, Sweden) and three different magnetic attachments were constructed according to the manufacturer's product data. Two implants were vertically oriented, mutually parallel, and 20 mm away from each other inserted in the bilateral mandibular canine region. The magnetic attachments consisted of a magnet, a keeper, and an abutment cylinder. The keeper (K) was screwed onto the abutment cylinder (A) and inserted into the implant, and the magnet (M) was embedded in the denture. Three different magnetic attachments were used in this research: flat-type (IP-DXFL; Aichi Steel Co., Japan), dome-type (IP-MCD; Aichi Steel Co., Japan), and cushion-type (IP-MCS; Aichi Steel Co., Japan) (Figure 3), divided as FM group, DM group, and CM group. The total numbers of elements and nodes of three models are listed in Table 1.

### 2.2. Material Properties and Interface Condition

The mechanical properties of the materials are presented in Table 2. The interface between implants and the bone was assumed to be absolute osseointegration [23]. The implant, the keeper, and the abutment cylinder were considered as a combination so that no motion among these structures occurs under applied loading [23]. To simulate the clinical situation that the overdenture was able to generate rotation and slide on the bottom mucosa in different directions when functioning, sliding friction contact was applied at the overdenture-mucosa interface, and the friction coefficient *μ* was set at 0.334 [15].

### 2.3. Constraints and Loading Conditions

**Figure 4:**
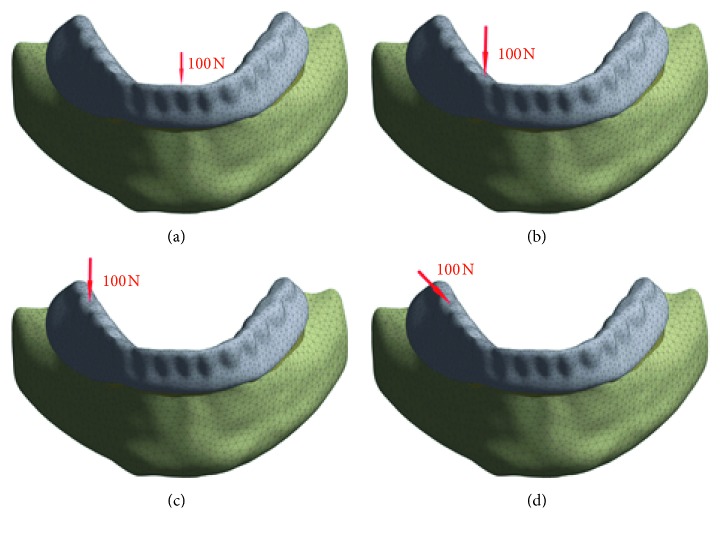
Four loading conditions. (a) Vertical load on the lower incisors (VI). (b) Vertical load on the right canine (VC). (c) Vertical load on the right first molar (VM). (d) Oblique load, 45° angled force buccolingually applied at the centre of the right first molar (OM).

The models were restrained at the nodes on the mandible within all directions in all degrees of freedom. To simulate the clinical masticatory loading, four types of 100 N load strength from different directions and positions were applied to the overdenture, namely, 100 N vertical load on the lower incisors, 100 N vertical load on the right canine, and 100 N vertical and oblique loads on the right first molar. The choice of a load with a magnitude of 100 N was based on the viewpoints that both the moderate level of biting force on implant overdentures and the average maximum occlusal force in complete denture patients were 100 N [24, 25]. The four loading conditions have been abbreviated as VI (vertical load on the lower incisors) (Figure 4(a)), VC (vertical load on the right canine) (Figure 4(b)), VM (vertical load on the right first molar) (Figure 4(c)), and OM (oblique load on the right first molar) (Figure 4(d)). OM refers to a 45° angled force buccolingually applied at the centre of the right first molar [15].

## 3. Results

### 3.1. Stress Distribution in Peri-Implant Cortical Bone

**Figure 5:**
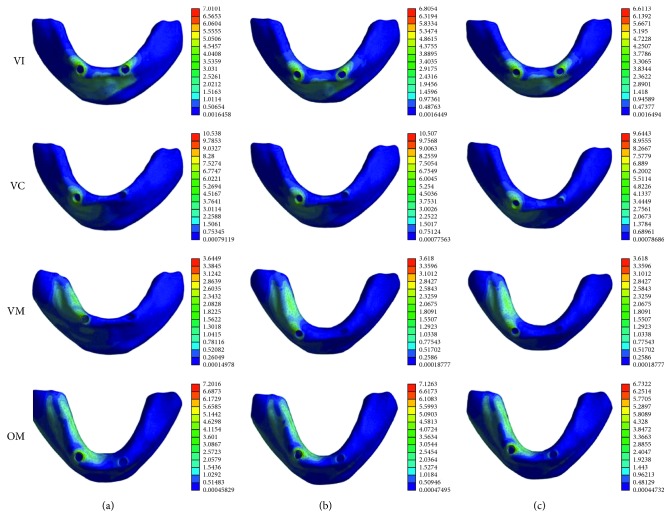
Maximum equivalent stress in the peri-implant bone of the flat-type model, dome-type model, and cushion-type model in four loading situations. Colors indicate level of stress from dark blue (lowest) to red (highest) (MPa). (a) Flat-type. (b) Dome-type. (c) Cushion-type.

Among the attachment types and loading conditions, the stress areas were mainly distributed around the loading side (Figure 5).

The FM group exhibited the highest levels of maximum equivalent stress in the peri-implant bone under VI, VC, and OM loading conditions, and the peak stress values in the cortical bone were shown in VC loading condition.

When the vertical load was applied on the right first molar, the maximum equivalent stress in the peri-implant cortical bone was much less than the vertical load applied on the incisor or on the canine. But when the vertical load changed to be oblique load, the maximum equivalent stress in the peri-implant cortical bone is about two times as the VM loading condition (Table 3).

### 3.2. Stress Distribution in Dental Implant

**Figure 6:**
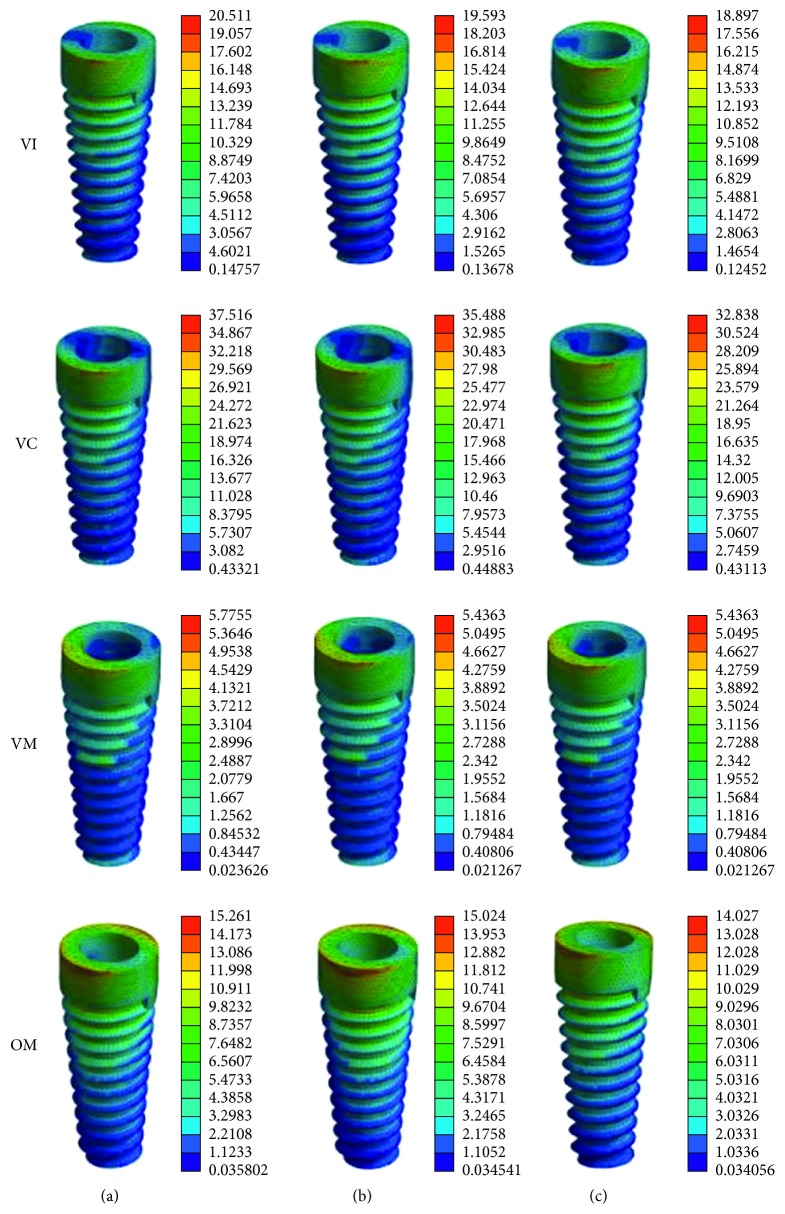
Maximum equivalent stress in dental implant of the flat-type model, dome-type model, and cushion-type model in four loading situations. Colors indicate level of stress from dark blue (lowest) to red (highest) (MPa). (a) Flat-type. (b) Dome-type. (c) Cushion-type.

The stress distribution in the dental implant showed a similar trend as in the peri-implant cortical bone (Figure 6). The peak maximum equivalents stress is in VC loading condition in the FM group. The lowest levels of maximum equivalents stress are in VM loading condition (Table 4).

### 3.3. The Pressure on the Mucosa

**Figure 7:**
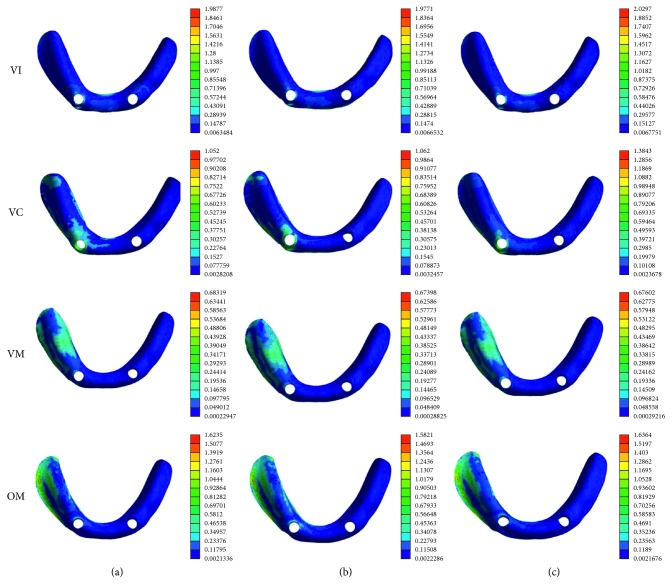
Maximum equivalent stress on the mucosa of the flat-type model, dome-type model, and cushion-type model in four loading situations. Colors indicate level of stress from dark blue (lowest) to red (highest) (MPa). (a) Flat-type. (b) Dome-type. (c) Cushion-type.

The maximum pressures on the mucosa were higher in VI, VC, and OM loading condition than in VM loading condition (Figure 7). In VM loading condition, the maximum pressure on the mucosa of FM group, DM group, and CM group was almost the same. When the vertical load changed to be oblique load, the maximum pressure on the mucosa is about two times as the VM loading condition. In the same loading condition, the CM group mostly showed the highest maximum mucosa pressures, and the peak maximum pressure was observed in the CM group in VI loading condition (Table 5).

### 3.4. The Deformation of the Mucosa

**Figure 8:**
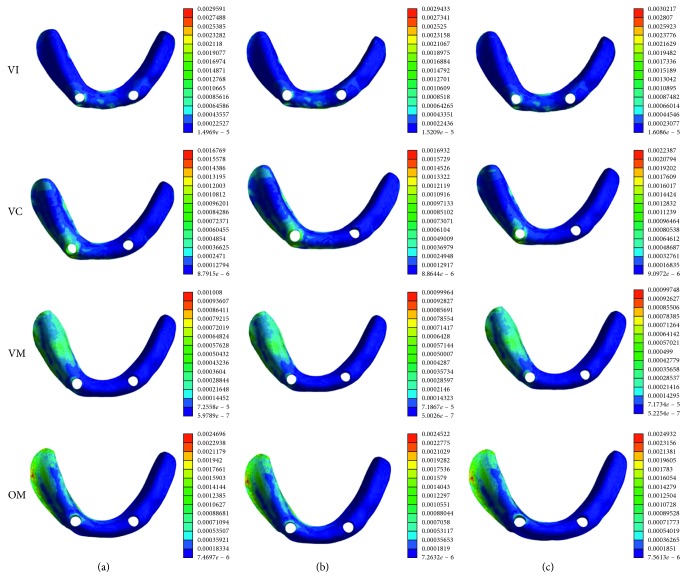
Distribution of the mucosa deformation of the flat-type model, dome-type model, and cushion-type model in four loading conditions. Colors indicate level of strain from dark blue (lowest) to red (highest) (10^−3^ *μ*m/*μ*m). (a) Flat-type. (b) Dome-type. (c) Cushion-type.

The maximum deformation of the mucosa showed a similar trend as the pressure on the mucosa (Figure 8). The lowest levels of maximum mucosa deformation are in VM loading condition. When the vertical load changed to be oblique load, the maximum mucosa deformation is about two times as the VM loading condition. The peak maximum deformation of the mucosa is in VI loading condition in the CM group. In the OM group, the peak deformation was concentrated in the distal border seal area (Table 6).

## 4. Discussion

Dental implants are used to stabilize complete mandibular dentures, and the two-implant-supported mandibular overdentures are considered to be the most economical and effective treatment for edentulous patients [26]. Previous studies have demonstrated that the retentive force of magnets is adequate to aid denture retention and provide patients with great satisfaction [27, 28]. Magnetic attachments which are shorter and do not follow a particular path of insertion compared to mechanical attachments can be used in edentulous patients, especially the cases of reduced interarch space or in moderately nonparallel abutments [29] or patients with physical disabilities for they are easy to place and remove [30]. The clinical study of Ellis et al. [27] indicated that more than 30% of patients prefer the magnetic attachment as the retention system within implant-supported mandibular overdentures for comfortable feeling and ease of cleaning. Meanwhile, Cheng et al. showed that implant-retained magnetic attachment can significantly improve the masticatory efficiency of mandibular overdenture, improve the comfort level, and greatly improve the satisfaction [28].

In VI, VC, and OM loading conditions, the flat-type model exhibited higher maximum equivalent stress in the peri-implant bone than dome-type and cushion-type models, which can be explained by the difference in the load transfer mechanism of various attachments. The flat-type attachment can provide the strongest retentive force, but the stress is easy to concentrate with a lack of resilience [31]. The dome-shaped type is manufactured to reduce the stress level by allowing the denture movement to a certain extent, while the cushion-shaped type is primary through the stress distributor effect of the elastic cushion pad [3]. Our data showed that the peak maximum deformation of the mucosa is in VI loading condition in the CM group. It demonstrated that under vertical force in the upward-downward direction, flexible cushion is helpful for transferring the force to oral mucosa to reduce the vertical force on the dental implant. Our data are highly consistent with the results of Takeshita et al. [14] that the characteristics of different attachment systems will affect the stresses generated in the peri-implant bone of mandibular overdenture. In terms of stress distribution, dome-type and cushion-type attachments may be a better choice to reduce the stress generated in the peri-implant bone during vertical loading condition.

The oblique force was applied buccolingually on the right first molar to simulate the chewing forces. It can be seen that the maximum stress on the peri-implant bone under oblique loading was approximately two times as those under vertical loading. Oblique load is thought to be harmful for stress distribution on the implants [32]. Our results indicated that the peri-implant bone damage is more likely happened under oblique loading than vertical loading. Compared with the flat-type attachment, dome-type and cushion-type attachments exhibit less stress on the peri-implant bone under oblique loading condition. This is due to the stress-breaking ability of these two attachments [33, 34]. The study by Gonda et al. [3] has demonstrated that the magnetic attachment with stress breaker caused lower lateral stress than conventional magnetic attachment. The effect of the cushion materials and allowance in rotational movement of the dome-shaped configuration are beneficial for mitigating the lateral stresses on the peri-implant bone, which may minimize traumatic loads towards the implant fixture.

In the implant-retained overdenture, the movement of the denture should also be considered [35]. Retention of the overdenture results from the type of the attachment system, and the pressure on the denture border sealing area affects the denture base coordination. In oblique molar loading condition, the highest maximum deformation of the mucosa was approximately two times as high as in VM loading conditions, and the deformations of the mucosa were mainly concentrated in the distal border seal area. It inferred that the oblique force leads to the largest deformation of the mucosa, which may ultimately destroy the denture border sealing effects. A previous report compared the stress distribution around implant and movement of overdentures retained with ball and three different types of magnetic attachments [11]. The authors concluded that magnetic attachments could be a better choice based on lower stress on peri-implant bone and better denture stability. Meanwhile, they also indicated that when the dentures were under too much lateral loads, the magnetic attachment was not stable. It is generally accepted that the low resistance to lateral forces is one of the greatest advantages of magnetic attachment, and the loss of retention under excessive oblique loading may help to protect the implant against unfavorable lateral forces, especially for patients with osteoporosis or when a shorter or smaller diameter implant has to be used due to bony deficiency.

Based on these results, it can be suggested that the selection of a magnetic attachment system for two-implant-retained overdentures should be carried with caution. In patients with osteoporosis or bony deficiency, dome-type or cushion-type attachments should be better choices than the flat-type attachment. From the official website of Aichi Steel Company (http://www.aichi-steel.co.jp), the flat-type magnetic attachment is indicated only for four-implant-supported overdentures. The limited usage of this system for two-implant-retained overdentures can be attributed to the relatively higher levels of lateral forces and strain/stress distribution when compared with the other two magnetic systems, which has been demonstrated by our FEA analysis.

Three-dimensional finite element method used in this study has some limitation in predicting the response of applied loadings [36–38]. First, the structures were considered isotropic, homogeneous, and linearly elastic, and perfect osseointegration between implants and bone was also hypothesized. Secondly, only one oblique force on the right first molar was applied to the model. In fact, the occlusal forces are multidirectional, so it is hard to simulate the complicate stress distribution. However, our data may provide a deeper understanding about the biomechanical behaviors of magnetic attachment. Long-term clinical studies are needed to assess the effects of different types of magnetic attachment on mandibular injuries and denture function.

## 5. Conclusions

Within the limitations of the study, the following conclusions were drawn:

1. Flat-type magnetic attachment exhibited higher levels of maximum equivalent strain/stress in the peri-implant bone compared to dome-type and cushion-type attachments under vertical and oblique loading conditions
2. Oblique loading may play a detrimental role for all magnetic attachments in strain/stress distribution and denture stability
3. Cushion-type and dome-type attachments are better choices in two-implant-retained mandibular overdentures, especially for patients with bad bone conditions such as osteoporosis or when a shorter or smaller diameter implant has to be used

## Figures and Tables

**Table 1:** Total number of elements and nodes.

	Elements	Nodes
Flat-type model	29,989	54,547
Dome-type model	30,429	55,211
Cushion-type model	50,487	90,314

**Table 2:** Material properties.

Material	Structure	Young's modulus (Mpa)	Poisson's ratio	Reference
POM	Cushing pad	5000	0.36	Manufacture^*∗*^
AUM20	The keeper (K)	200,000	0.28	Manufacture^*∗*^
Ti-6Al-4V	Implant	110,000	0.33	Colling [17]
NdFeB (magnet)	The magnet (M)	160,000	0.24	John et al. [18]
Pure titanium	The abutment cylinder (A)	117,000	0.30	Sakaguchi and Borgersen [19]
Acrylic resin	Artificial teeth and denture base	8300	0.28	Darbar et al. [20]
	Cortical bone	13,700	0.3	Barbier et al. [21]
	Trabecular bone	1370	0.3	Barbier et al. [21]
	Oral mucosa	680	0.45	Barao et al. [22]

^*∗*^Personal communication.

**Table 3:** Maximum equivalent stress in the peri-implant bone (MPa).

Loading condition	Flat-type model	Dome-type model	Cushion-type model
VI	7.0701	6.8054	6.6113
VC	10.538	10.507	9.6443
VM	3.644	3.618	3.688
OM	7.202	7.127	6.7322

**Table 4:** Maximum equivalent stress in dental implant (MPa).

Loading condition	Flat-type model	Dome-type model	Cushion-type model
VI	20.511	19.593	18.897
VC	37.516	35.488	32.838
VM	5.775	5.436	5.726
OM	15.261	15.026	14.027

**Table 5:** Maximum equivalent stress on the mucosa (MPa).

Loading condition	Flat-type model	Dome-type model	Cushion-type model
VI	1.987	1.9771	2.0297
VC	1.052	1.062	1.3843
VM	0.683	0.673	0.676
OM	1.623	1.582	1.6364

**Table 6:** The maximum deformation of the mucosa (10^−3 ^*μ*m/*μ*m).

Loading condition	Flat-type model	Dome-type model	Cushion-type model
VI	5.72	5.55	5.62
VC	6.66	6.49	6.58
VM	4.40	4.16	4.16
OM	18.05	17.83	16.59

## Data Availability

The data used to support the findings of this study are included within the article.

## Abbreviations

VM: 100 N vertical load applied on the right first molar
VC: 100 N vertical load applied on the right canine
VI: 100 N vertical load applied on the lower incisors
OM: 100 N oblique load applied on the right first molar.
